# Evaluating the operational resilience of small and medium-sized enterprises to flooding using a computational modelling and simulation approach: a case study of the 2007 flood in Tewkesbury

**DOI:** 10.1098/rsta.2019.0210

**Published:** 2020-02-17

**Authors:** Graham Coates, Meshal Alharbi, Chunhui Li, Sangaralingam Ahilan, Nigel Wright

**Affiliations:** 1 School of Engineering, Newcastle University, Newcastle-upon-Tyne NE1 7RU, UK; 2 Department of Computer Science, Durham University, Durham DH1 3LE, UK; 3 School of Civil Engineering, University of Leeds, Leeds L62 9JT, UK; 4 School of Architecture, Design & Built Environment, Nottingham Trent University, Nottingham NG1 4FQ, UK

**Keywords:** small and medium-sized enterprises, resilience, flood modelling, agent-based modelling

## Abstract

The resilience of small and medium-sized enterprises (SMEs) to disruptive events is significant as this highly prevalent category of business forms the economic backbone in developed countries. This article provides an overview of the application of a computational modelling and simulation approach to evaluate SMEs' operational resilience to flooding based on combinations of structural and procedural mitigation measures that may be implemented to improve their premises' resistance to flooding and safeguard their business continuity. The approach integrates flood modelling and simulation with agent-based modelling and simulation (ABMS) within a modelled geographical environment. SMEs are modelled as agents based on findings of semi-structured interviews with SMEs that have experienced flooding or are at risk of flooding. In this paper, the ABMS has been applied to a new case study of the major flood event of 2007 in Tewkesbury. Furthermore, to enable an evaluation of the operational resilience of manufacturing SMEs in terms of the relative effectiveness of flood mitigation measures, a new coefficient based on production loss is introduced. Results indicate structural mitigation measures are more effective than procedural measures. While this result is intuitive, the approach provides a means of evaluating the relative effectiveness of combinations of mitigation measures that SMEs may implement to enhance their operational resilience to flooding.

This article is part of the theme issue ‘Urban flood resilience’.

## 1. Introduction

Business and organizational resilience to disruptions, disturbances and discontinuities is an evolving area of research [1–7]. However, in both a business context and more generally, a consensus has yet to be reached regarding the meaning of the term *resilience* with many descriptions existing. For example, *resilience* has been defined as the adaptation ability of an organization to return to a stronger state post-disturbance [7], the amount of disturbance a system is able to absorb and still remain within the same state [8], and as the ability to maintain a stable state in the face of external shocks and disturbances [9]. In particular, a growing body of research has emerged in relation to the resilience of small and medium-sized enterprises (SMEs) [10–13] with a number of researchers focusing on resilience and recovery to extreme weather events [14,15] and flooding [16–19]. In spite of this emerging body of research, it has been asserted that the impact of natural disasters on small businesses has been understudied [20]. Similarly, with a particular emphasis on flooding, it has been indicated that there is a dearth of research involving businesses, preparedness and recovery [21] and its impact upon small businesses is largely unexplored [22].

In relation to natural disasters, including extreme weather events, it has been recognized that small businesses need to proactively prepare for such situations [23] and become more resilient to them [12]. In terms of flooding, it has been highlighted that as climate change makes these events more frequent, businesses will need to better prepare to respond to this threat [24]. Despite the calls for SMEs to have greater resilience and better preparedness to disruptive events, it is widely acknowledged these businesses are generally not prepared for natural hazards such as flooding [25] and remain highly vulnerable to extreme weather events [15]. In the light of these acknowledgements, a need is identified to investigate SMEs' coping strategies in facing up to extreme weather events, including flooding, and increasing their resilience to them. Relatedly, a recent review considers strategies to help develop the resilience of SMEs in the face of adversity and identifies the need to deliver practical guidance for SMEs endeavouring to be more resilient [26].

In addition to SMEs being recognized as lacking organizational resilience to disruptive events [27], a reason for the focus of research on the resilience of these small businesses relates to a comparison with their larger counterparts. That is, SMEs have limited resources [28], no organizational slack [29] and lack continuity plans [30], all of which contribute to them being under-prepared for a major disruptive event [31]. Consequently, SMEs' recovery and return to normalcy, if possible, can be a lengthy process. Another important reason for SMEs becoming a focal point of research in the context of resilience to disruptive events is that they represent the most significant share of businesses in most developed countries. For example, in the UK, SMEs represent 99.9% of businesses, account for 60% of employment and 52% of turnover [32]. Furthermore, in the UK, SMEs are divided into three categories according to number of employees: micro-sized businesses have up to nine employees, small-sized have 10–49 employees and medium-sized 50–249 employees. Micro-sized businesses represent the most significant of these categories, accounting for 96% of all businesses in the UK. Given the prevalence and thus importance of SMEs, any disruption to the continuity of their business operations can have a major impact on the national economy. Indeed, given small businesses are essential to the economy, it has been emphasized that it is important to gain a greater understanding of their recovery from natural disasters [20]. Consequently, there is a need to better understand how this important type of business can be made more resilient to disruptive events such as flooding, which has been recognized as a key risk in many parts of the UK. That is, in the National Risk Register of Civil Emergencies [33], river (fluvial) and coastal flooding are rated 3 out of 5 as likely to occur in the next 5 years with an impact severity of 4 out of 5. Similarly, surface water flooding is rated as having the same likelihood of occurring but with an impact severity of 3 out of 5.

In many domains, computational modelling and simulation offers an inexpensive and time-efficient means of carrying out what-if scenarios and investigations into alternative strategies for a given situation. In particular, agent-based modelling and simulation (ABMS) is one of the most prominent computational approaches used, which enables investigations into how the dynamics of a real-world system are likely to be affected by changes to internal or external factors [34]. Furthermore, ABMS is a bottom-up approach in which the behaviour of a system can be studied via modelling and simulating the behaviours and interactions of individual agents that represent system entities such as people, organizations and businesses. ABMS has been applied in many fields at an increasing rate in the past decade [35,36]. In relation to flooding, applications of agent-based models include flood evacuation simulation [37], risk-based flood incident management [38], flood risk and insurance [39], flood risk communication strategies [40], surface water flood risk and management strategies [41], evolving community flood risk [42], social media and individual behaviours in flood evacuation processes [43] and flood loss assessment with household responses [44]. In the context of SMEs, agent-based models have been used in applications such as dynamic re-scheduling [45], mechatronic supply chains [46], public policy impact [47], community impact on resilience [48], cost collaborative management in supply chains [49], collaboration duration on supply chains [50] and dynamic supply chain formation [51]. Given the widespread use and applicability of agent-based modelling and simulation, in this research, it has been selected to be coupled with flood modelling and simulation to facilitate evaluations of structural and procedural mitigation measures that may be implemented by SMEs in order to become more resilient to extreme inundation events.

In this paper, to follow, the methods employed in the computational modelling and simulation approach used to evaluate SMEs' operational resilience to flooding are summarized and the case study to which the approach has been applied is presented. In the approach, a modelled geographical environment provides the common physical environment for the simultaneous modelling and simulation of a flood event and SME operations before, during and after this event. The case study of the flooding experienced in Tewkesbury, Gloucestershire, was selected due to the extreme nature of the event and its impact in this urban area on manufacturing SMEs. Prior to presenting and discussing the results obtained by applying the approach, simulation experiments in terms of flood mitigation measures that SMEs can implement are defined and a measure, based on production capacity loss, is proposed to enable an evaluation of manufacturing SME operational resilience. Finally, conclusions of the research are summarized and an indication of the direction of future work is given.

## 2. Methods

**Figure 1.**
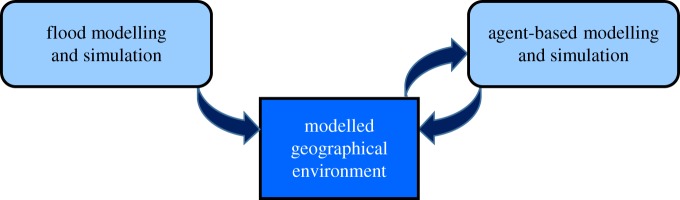
Computational modelling and simulation approach. (Online version in colour.)

In this research, computational modelling and simulation forms the approach to enable an evaluation of SMEs' operational resilience to flooding. Figure 1 provides an overview of the approach in which modelling and simulation of a flood event is pre-computed and then fed into a modelled geographical environment at regular intervals (one simulation tick represents 30 min) throughout the specified time period of the agent-based simulation.

### (a) Geographical environment modelling

**Figure 2.**
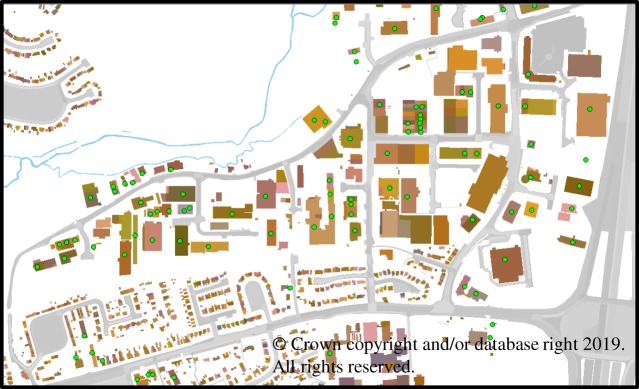
Modelled geographical environment with SMEs' premises indicated using green circles. (Online version in colour.)

A model of the geographical environment of the urban area under consideration is required as it provides a common physical environment in which to simultaneously perform flood event simulation and agent-based simulations of SMEs' operations. The modelled geographical environment has been constructed using data from three layers of the ordnance survey (OS) MasterMap^©^, namely the Topography layer, Integrated Transport Network™ layer and Address layer. Each of these layers includes geographical information systems (GIS) data, which can be represented visually as shown in figure 2.

In figure 2, the modelled geographical environment, including buildings and the road network, is shown for part of Tewkesbury in Gloucestershire, which is the location of the case study presented in §3. Also, in figure 2, buildings that form the premises of SMEs are indicated using green circles. Using OS MasterMap's Address layer, not only can SMEs' premises be identified, but also the name and sector of each of these businesses.

In the geographical area of Tewkesbury considered in §3, 692 SMEs were identified, 92 of which were manufacturing SMEs according to the Address layer from OS MasterMap^©^. Furthermore, 16 of these 92 manufacturing SMEs were directly affected by the flood event modelled and simulated. Thus, these 16 manufacturing SMEs, assumed to be micro-sized given their dominance, were the focus of the agent-based simulation experiments defined in §4 and discussed in §5.

### (b) Flood modelling and simulation

Flood modelling and simulation involved using linked one-dimensional (1D) and two-dimensional (2D) models within the LISFLOOD-FP hydrodynamic model, which was developed at Bristol University [52]. In LISFLOOD-FP, the 1D-model represents the river channel using a kinematic approximation of the 1D Saint-Venant equations solved using an implicit Newton–Raphson scheme. The 2D-model represents flow in the urban environment located on the floodplain using the full 2D shallow water equation solved using the finite volume approach [53]. The 1D and 2D models in LISFLOOD-FP are dynamically linked, which allows flow data to be exchanged between the river channel and floodplain [54]. Flow is defined as the volume of water transferred through a specific section of the river per unit time. In the LISFLOOD-FP hydrodynamic model, much attention has been paid to linking the 1D and 2D models to achieve a good representation of both channel and floodplain conveyance of rivers. The hydrodynamic model has four input datasets: hydrographs, OS MasterMap^©^'s Topography Layer, river survey data and Digital Terrain Model (DTM) data. Hydrographs provide a time series of flow (discharge) over the duration of a flood. In this study, measured hydrographs of the River Severn and River Avon were used for input to the LISFLOOD-FP 1D model upstream of the geographical urban area of Tewkesbury under consideration. OS MasterMap^©^'s Topography Layer (scale 1:1250) provides building polygon and land use data allowing the assignment of no-flow areas around buildings and the application of different surface roughness (based on their resistance to flow) to the land use classes in the LISFLOOD-FP 2D model. Buildings were considered to be a constant 5 m above local ground-level elevation. River survey data provide information regarding the elevation of the river channel in the LISFLOOD-FP 1D model; this was obtained from the existing ISIS 1D model provided by the Environment Agency and was used to represent the river channel in the 1D model. In the LISFLOOD-FP 2D model, the DTM datasets from the Environment Agency were used to represent the topography of the floodplain. The DTM was produced from the light detection and ranging (LiDAR) datasets obtained from an airborne mapping technique that uses a laser to measure the distance between the aircraft and the ground. The DTM represents elevation of the floodplain at regular grid intervals of 2 m. Finer resolution datasets are available; however, these provide only a small increase in accuracy and their use is computationally intensive in simulations of longer duration rainfall events in the LISFLOOD-FP model.

### (c) Agent-based modelling and simulation

Agent-based modelling has been used to represent SMEs and associated organizations, such as suppliers and customers, and the relationships between them in the context of a flood event. Furthermore, agent-based simulations, allied with flood simulation, have been performed to evaluate the effect of implementing flood mitigation measures on the operational resilience of SMEs to an extreme inundation event. Behaviours of agents representing SMEs have been obtained primarily from transcripts of semi-structured interviews with over one hundred SMEs from across the UK that have experienced flooding or are at risk of flooding. That is, a detailed analysis of these transcripts was undertaken manually, which revealed a variety of behaviours that SMEs may exhibit before and after a flood event. Furthermore, the conditions that would need to be in place to cause an SME to exhibit particular behaviours were gleaned from the interview transcripts along with indications of the interdependencies between behaviours. In addition, the typical duration range of each behaviour was established based on statements made by SME employees in the interview transcripts. For example, pre-flood, an SME receiving Environment Agency warnings may take action to reduce the possible damage and disruption caused by flood water entering its premises by moving equipment and raw materials to ‘safer' locations, which would prevent them from being affected. Post-flood, an SME may interact with their insurance company, suppliers, customers and other organizations that will assist in the company's recovery. Details of SME attributes and behaviours modelled, and the relationships between them, can be found in [55]. In addition, an overview of the validation approach followed for the ABMS is discussed in terms of conceptual and operational validation, which involved analysing transcripts from over one hundred interviews with SMEs and running two SME workshops in Tewkesbury and Sheffield. By contrast with the research presented in [55], this article includes an application of ABMS to a new case study, i.e. the significant 2007 floods in Tewkesbury, and introduces a new operational resilience measure to enable the relative effectiveness of flood mitigation measures implemented by manufacturing SMEs to be evaluated.

In an agent-based simulation, for each manufacturing SME at each tick (representing a 30 min period), production capacity is determined based on the proportion of available employees and machines, the current level of raw materials, and whether or not the premises has power; note that all machines are assumed to be located at ground floor level. That is, at any simulation tick, if all employees and machines are available, sufficient raw materials are in stock to manufacture products, and power is being supplied to a manufacturing SME's premises, then production capacity is 100%. This level of production capacity is expected pre-flood, until employees evacuate the premises or unless they are relieved from production operations and used to carry out preventative actions to reduce potential damage. By contrast, during the flood event, production operations are suspended or not possible meaning production capacity is zero. Post-flood, at some point during recovery, production will be resumed and capacity increases from zero until it eventually reaches the pre-flood level of 100%, typically following a nonlinear profile as a function of the availability of employees and machines, raw material levels, and whether or not the premises has power. For each manufacturing SME, its ‘recovery' profile will be influenced to some degree by the actions undertaken pre-flood, but most significantly by the flood mitigation measures put in place aimed at preventing or minimizing the level of flood water entering its premises and the disruption to business continuity. In the agent-based simulations performed, flood mitigation measures have been categorized as structural or procedural. A list of these measures is given in §4, in which a number of simulation experiments are defined and the procedure for determining an operational resilience coefficient as a function of loss in production capacity is outlined.

## 3. Case study: Tewkesbury floods of 2007

**Figure 3.**
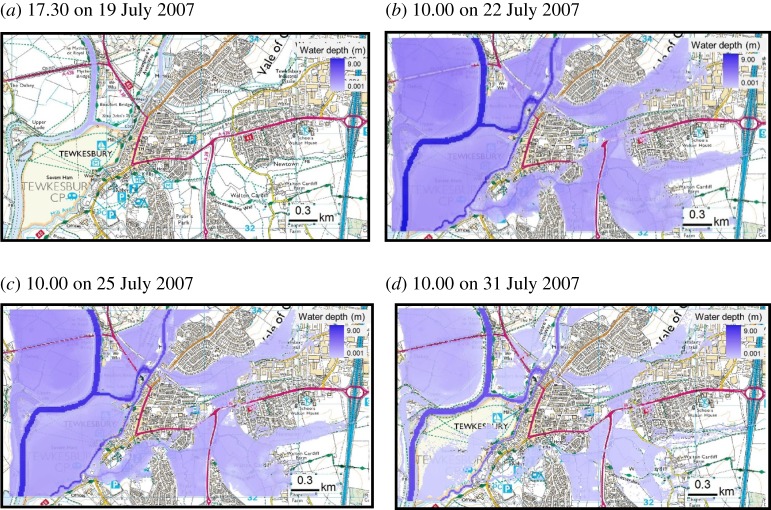
(*a*–*d*) Flood modelling and simulation in Tewkesbury, Gloucestershire. (Online version in colour.)

In South West England, on 20 July 2007, two months' worth of rain fell in 14 h resulting in widespread flooding of Tewkesbury in Gloucestershire. Tewkesbury is vulnerable to flooding due to its location relative to two rivers, the Severn and the Avon, which meet in the town. Flooding lasted for approximately two weeks severely affecting the town through a combination of pluvial and fluvial flooding. Due to the large proportion of highly developed areas in Tewkesbury, a significant proportion of the rainfall resulted in fast run-off. Also, the heavy rainfall overwhelmed the drainage and sewerage systems, which led to localized pluvial flooding in the town. The prolonged intense rainfall event led to unprecedented levels of flooding as the rivers Avon and Severn burst their banks in many places on 21 and 22 July. Representations of the geographical area of Tewkesbury pre-flood are shown in figure 3*a* and during the inundation event based on output from flood modelling and simulation in figure 3*b*–*d*. Furthermore, figure 3*b*–*d* provides an indication of the extent of the flooding at various times.

In the flood modelling and simulation of the extreme flood event of July 2007 in Tewkesbury, the LISFLOOD-FP hydrodynamic model was used to simulate flow propagation using the measured hydrographs of the River Avon and River Severn. Furthermore, within the hydrodynamic model, the land use and river topography datasets enabled the establishment of flow pathways in the rivers and the town of Tewkesbury. The flood was simulated from 17.30 on 19 July to 23.30 on 1 August, with the flood inundation steadily increasing and peaking on 22 July. In terms of output, the model predicted flood depth, flow velocity and inundation period. On 22 July, flood depths of up to 8.5 m in the river channel and over 3 m in the town were predicted by the simulation. As the flood gradually receded in the latter part of July, Tewkesbury essentially became an island, particularly in the High Street area. Throughout the two-week period flood simulation, water depths were recorded at half-hour intervals at all easting and northing locations within the modelled geographical environment study area. Given the modelling of this environment involved the use of the Address layer of OS MasterMap**^©^**, a one-to-one mapping was enabled between the water depth at each location as it varied throughout the time frame of the flood event modelled and the precise locations of all SMEs, including the manufacturing SMEs focused on in the case study area of Tewkesbury.

In terms of flood model validation and calibration, the approach taken involved comparing the simulated water level obtained from the model with the measured/observed water level at Mythe Bridge in Tewkesbury over a 30-day period from 22.00 on 11 June 2007. That is, using the model, in line with standard practice, the adjustment of the channel roughness and floodplain roughness parameters was varied until the output agreed with over 90% of the observed water level measurements at Mythe Bridge. It is highlighted that data scarcity is a challenge for flood model validation. Thus, the next steps for validation of the flood model require more data from actual flood events, for example, via remote sensing.

## 4. Simulation experiments and SME operational resilience

### (a) Simulation experiments

To evaluate manufacturing SMEs' operational resilience to an extreme flood event, seven agent-based simulation experiments have been defined and performed. These simulation experiments have been defined to evaluate the effect of different sets of structural and/or procedural flood mitigation measures that an SME can put in place to improve their operational resilience to flooding. Structural measures are physical changes made to an SME's premises to prevent water inundation or lessen the damage should this happen. By contrast, procedural measures are those actions and activities an SME can undertake to safeguard business continuity as far as is possible. Table 1 defines the seven agent-based simulation experiments undertaken in relation to the structural and procedural flood mitigation measures listed. Furthermore, in table 1, it can be seen that both types of flood mitigation measures are sub-categorized; physical measures can be easy or harder to implement whereas procedural measures can be internal or external to an SME.

**Table 1.** Agent-based simulation experiments.

		experiment (E)						
flood mitigation measures		1	2	3	4	5	6	7
structural (easy to implement)								
S1	raise the level at which paper documents are kept		✓	✓				✓
S2	raise the level at which raw materials are stored		✓	✓				✓
S3	raise the level at which products are stored		✓	✓				✓
S4	keep sandbags on site to seal doorways		✓	✓				✓
S5	paint exterior of building with waterproofing substances, deploy floodgates and airbrick covers		✓	✓				✓
structural (harder to implement)								
S6	maintain an electricity generator			✓				✓
S7	raise the level at which machines are located			✓				✓
S8	raise the level at which electrical sockets and consumer boards are located			✓				✓
S9	install flood-resilient flooring			✓				✓
S10	install anti-backflow valves			✓				✓
S11	maintain/use sump pumps			✓				✓
procedural (internal)								
P1	store all documentation electronically and maintain backups of them				✓		✓	✓
P2	prepare a package of contacts (e.g. customers, suppliers, insurance company, contactors)				✓		✓	✓
P3	prepare and maintain an emergency plan for business continuity				✓		✓	✓
P4	perform emergency flood exercises				✓		✓	✓
P5	display flood plan instructions				✓		✓	✓
P6	maintain an emergency financial reserve				✓		✓	✓
procedural (external)								
P7	register for environment agency flood alerts and warnings					✓	✓	✓
P8	hold comprehensive insurance cover					✓	✓	✓
P9	have pre-existing mutual aid partner(s)					✓	✓	✓
P10	identify mutual aid partner(s)					✓	✓	✓
P11	request mutual aid					✓	✓	✓

In table 1, experiment 1 corresponds to all manufacturing SMEs not having implemented any of the flood mitigation measures, and thus provides a benchmark for the remaining experiments. Experiment 2 involves all SMEs putting in place structural measures considered easy to implement, whereas experiment 3 includes both easy and harder to implement measures. Note that an experiment in which only harder to implement structural measures are considered has not been defined since it is viewed that if an SME could put in place these measures then it would also implement the easy measures. Experiments 4–6 involves all SMEs having procedural measures classified as internal, external or both, respectively. Finally, experiment 7 involves all manufacturing SMEs having all structural and procedural flood mitigation measures in place.

### (b) SME operational resilience

**Figure 4.**
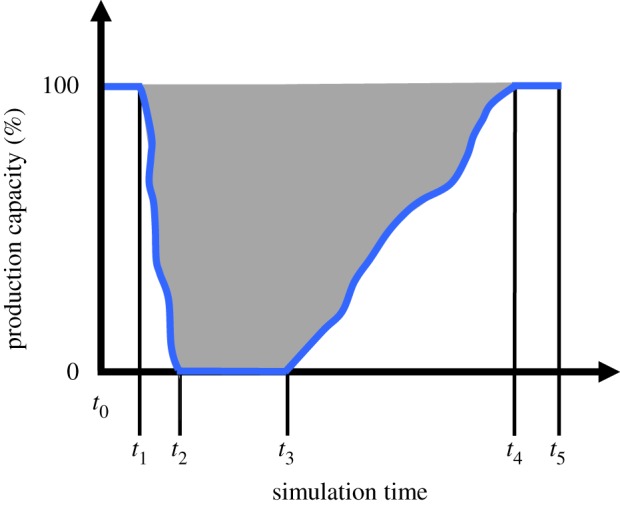
Profile of a manufacturing SME's production capacity pre- during and post-flood. (Online version in colour.)

In this research, the concept of operational resilience is inspired by the resilience triangle [56] and extension thereof [57]. A visualization of the profile of a manufacturing SME's production capacity over the simulation period, *t*_5_, is shown in figure 4. Pre-flood, from the start of the simulation period up to *t*_1_, production capacity is 100% assuming all employees and machines are available, sufficient raw materials are in stock to manufacture products and power is being supplied to the manufacturing SME's premises. The period *t*_1_–*t*_2_ corresponds to the time during which an SME's employees may take action to prepare for the flood event that may start at or after *t*_2_ and end before *t*_3_. In figure 4, from *t*_1_ to *t*_2_, the SME's production capacity is shown to decrease nonlinearly from 100% to zero. Note that if an SME were to take no preparatory action due to employees evacuating the premises immediately, then production capacity would drop from 100% to zero in a single simulation tick. From *t*_2_ to *t*_3_, which encompasses the flood event, the SME's production capacity is zero. Post-flood, the SME will undertake actions that bring about a resumption in production capacity at *t*_3_. From this time until *t*_4_, the SME's production capacity recovers until it reaches 100%. Again, the SME's production capacity is shown to vary (increase) nonlinearly, depending on actions taken pre- and post-flood, and the flood mitigation measures put in place.

In figure 4, the shaded area signifies the production capacity loss, $\text{P}{\text{C}}_{\text{loss}}$, as a direct result of the flood event. Mathematically, this can be expressed as equation (4.1).

4.1 $$\text{P}{\text{C}}_{\text{loss}}=100(t_{4}-t_{1})-\int_{t_{1}}^{t_{2}}f_{pre}(t)dt-\int_{t_{3}}^{t_{4}}f_{\text{rec}}(t)dt.$$

In equation (4.1), the two integrals signify the nonlinearity of the functions from *t*_1_ to *t*_2_ and *t*_3_ to *t*_4_. The first integral is associated with the pre-flood preparatory actions of a manufacturing SME whereas the second integral corresponds to the post-flood recovery actions undertaken. Also, flood mitigation measures taken by an SME will influence the impact of a flood event in terms of damage to its premises and disruption to its operations. That is, flood mitigation measures will influence the characteristics of the lines from *t*_3_ to *t*_4_.

In consideration of equation (4.1) and figure 4, it can be deduced that the greater the value of the two integrals, the lower the production capacity loss. In this research, a numerical integration method is used to approximate the definite integrals. Specifically, the trapezium rule is used to approximate a definite integral

4.2 $$J=\int_{a}^{b}f(t)dt,$$

where the interval a≤t≤b is subdivided into *n* sub-intervals of equal length Δt=(b−a)/n. Furthermore, in each sub-interval, the function f(t) is approximated by a trapezoid such that the area under the curve of f(t) between *a* and *b* can be expressed as

4.3 $$J=\Delta t\left[\frac{1}{2}f(a)+f(a+\Delta t)+f(a+2\Delta t)+\cdots +f(a+(n-1)\Delta t)+\frac{1}{2}f(b)\right].$$

In relation to equation (4.1), which is used to determine production capacity loss, an operational resilience coefficient, $\text{O}{\text{R}}_{c}$, can be established as stated in equation (4.4).

4.4 $$\text{O}{\text{R}}_{c}=1-\left(\frac{\text{P}{\text{C}}_{\text{loss}}}{100t_{5}}\right).$$

## 5. Results and discussion

**Figure 5.**
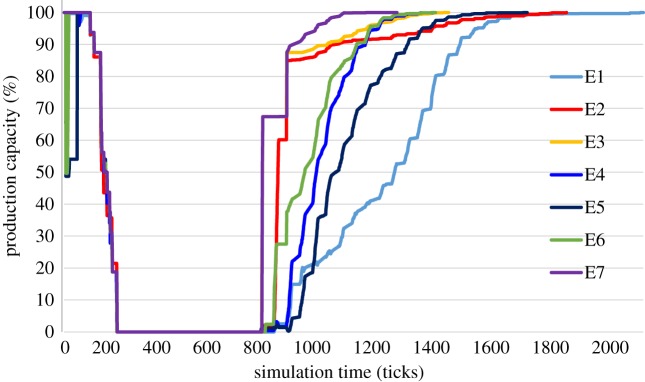
Average production capacity of 16 flood-affected manufacturing SMEs. (Online version in colour.)

Each agent-based simulation experiment defined previously in table 1 was performed 20 times. Based on these simulations, the average production capacity was calculated at each simulation tick taking into account all 16 flood-affected manufacturing SMEs modelled. For all seven simulation experiments, each of which is associated with different combinations of structural and/or procedural flood mitigation measures, figure 5 presents average production capacity, expressed as a percentage, for all 16 flood-affected manufacturing SMEs over the period of the simulation.

In figure 5, it can be seen that the most expeditious recovery post-flood corresponds to experiment 7, in which manufacturing SMEs have implemented all flood mitigation measures. By contrast, the slowest recovery is observed for experiment 1, in which SMEs have not put in place any mitigation measures. For the remaining five experiments, figure 5 provides a visual comparison of the recovery profile of the flood-affected manufacturing SMEs. Most of the recovery profile for experiment 2 (with easy to implement structural measures) is similar to that of experiment 3 (with easy and harder to implement measures). However, the latter part of the recovery is faster for experiment 3 with 100% production capacity being restored after 1402 ticks whereas for experiment 2 this occurs after 1842 ticks, i.e. approximately 9 working days later. For the experiments involving procedural flood mitigation measures, the recovery for experiment 6 (with internal and external procedural measures) is faster than that for experiment 4 (with internal procedural measures), which in turn is faster than that for experiment 5 (with external procedural measures).

The variations in the ‘recovery' profile of manufacturing SMEs for each experiment as seen in figure 5 are dependent on the effectiveness of the mitigation measures implemented and, in addition, the actions carried out by their employees post-flood. For example, if structural measures such as raising the level at which machines are situated prevents them from being damaged, then employees would not be required to repair them, meaning they could be deployed to carry out other actions. Similarly, if raising the level at which electrical sockets and consumer boards are situated stops them from being damaged, then there would be no need to call electricians to attend the SME's premises to undertake the necessary repairs to restore power. In summary, flood mitigation measures have the effect of influencing what a manufacturing SME must do post-flood and how long these actions will take before the business is able to resume production.

In all experiments, the simulation time *t*_5_ is taken as that recorded in the experiment with the greatest time taken for manufacturing SMEs to resume 100% production capacity. That is, in experiment 1, which is as expected, since in this case SMEs implemented none of the flood mitigation measures. Specifically, *t*_5_ corresponds to simulation tick 2124, which equates to a simulation duration of 1062 h or 44 working days and 6 h. Thus, for experiment 1, the time *t*_4_ and *t*_5_ are the same. Taking the same simulation duration for all experiments enables a direct comparison to be made between them in terms of the average production capacity loss of all 16 flood-affected manufacturing SMEs and the associated average operational resilience coefficient.

Table 2 presents output generated from all simulation experiments. In addition to average production capacity loss and the average operational resilience coefficient, table 2 includes information regarding the simulation timeline for each experiment.

**Table 2.** Simulation experiment output including operational resilience coefficients.

experiment	1	2	3	4	5	6	7
*t*_4_ (ticks)	2124	1842	1402	1353	1683	1348	1108
*t*_4_ (dd:hh)	44:06	38:09	29:05	28:05	35:02	28:02	23:02
*t*_4_*–t*_1_ (ticks)	2030	1748	1308	1259	1589	1254	1014
*t*_3_*–t*_2_ (ticks)	536	529	532	536	529	545	533
*t*_4_*–t*_3_ (ticks)	1395	1120	677	624	961	610	382
PC_loss_ (units)	102 192	70 107	63 629	79 146	87 846	74 603	61 398
OR*_c_*	0.519	0.670	0.700	0.627	0.586	0.649	0.711

**Figure 6.**
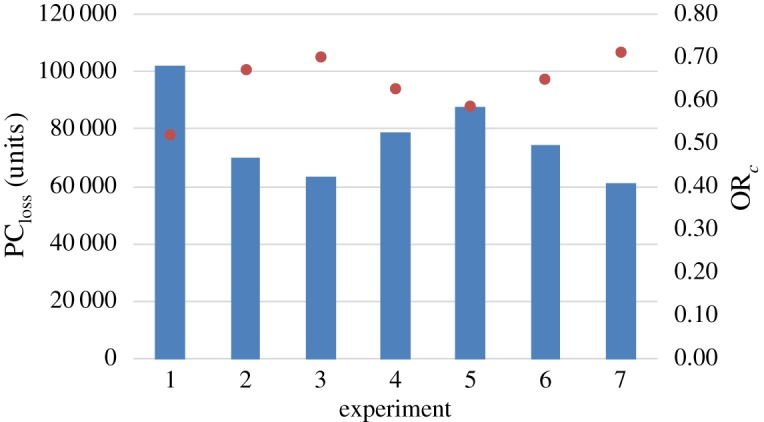
PC_loss_ (units) and OR*_c_* for each simulation experiment. (Online version in colour.)

In consideration of figure 4, recall that *t*_4_ corresponds to the time at which manufacturing SMEs resumed 100% production capacity; in table 2, these are shown in ticks, and working days and hours. As expected, experiment 1, with SMEs having no flood mitigation measures in place, resulted in the longest time to restore the pre-flood level of 100% production capacity. By contrast, experiment 7, with SMEs having implemented all structural and procedural flood mitigation measures, led to the least amount of time taken to restore 100% production capacity. In table 2, differences between particular times in the simulation period for each experiment are shown; figure 6 gives a visual representation of the variation in PC_loss_ (bar charts) and OR*_c_* (red dots) for each simulation experiment. In relation to table 2, the period *t*_4_–*t*_1_ corresponds to the time at which manufacturing SMEs' production capacity is first reduced, due to employees being occupied with taking preparatory actions or the flood water reaching their premises and/or employees evacuating the premises, through to the point post-flood when those businesses restore 100% production capacity. The period *t*_3_–*t*_2_, which includes the duration of the flood event, corresponds to the time during which SMEs have zero production capacity. The post-flood period *t*_4_–*t*_3_ corresponds to the time at which production capacity is resumed through to it reaching 100%.

In table 2 and figure 6, it can be seen that experiment 1, in which manufacturing SMEs implemented none of the flood mitigation measures, results in the greatest $\text{P}{\text{C}}_{\text{loss}}$, 102 192 units, and thus the lowest $\text{O}{\text{R}}_{c}$ of 0.519. Conversely, experiment 7, in which SMEs have all structural and procedural flood mitigation measures in place, yields the lowest $\text{P}{\text{C}}_{\text{loss}}$, 61 398 units, and therefore the greatest OR_*c*_ of 0.711. The difference between the operational resilience coefficients for these two experiments is 0.192, which is viewed as relatively small and is clearly related to both the severity and duration of the flood event considered. As expected, a comparison of values of $\text{O}{\text{R}}_{c}$ for experiments 2 and 3 confirms that having both easy and harder to implement structural mitigation measures, as opposed to only those easy to implement, provides an SME$\text{P}{\text{C}}_{\text{loss}}$with greater operational resilience to flooding. On comparing values of $\text{O}{\text{R}}_{c}$ for experiments 4 and 5, internal procedural mitigation measures are shown to lead to more resilient SMEs than external measures. Also, as expected, a comparison of experiments 4–6 confirms that having both internal and external procedural mitigation measures, as opposed to only internal or external, provides greater operational resilience to flooding. Experiments 3 and 6 demonstrate that structural flood mitigation measures offer greater operational resilience than procedural measures. In summary, a comparison of operational resilience coefficients in table 2 reveals the following relationships, ${\text{OR}}_{c}^{E7}>{\text{OR}}_{c}^{E3}>{\text{OR}}_{c}^{E2}>{\text{OR}}_{c}^{E6}>{\text{OR}}_{c}^{E4}>{\text{OR}}_{c}^{E5}>{\text{OR}}_{c}^{E1}.$

In order to determine if the means of one experiment output data are significantly different from another, a paired *t*-test was carried out at two points in time during the agent-based simulation for each experiment, namely in the recovery period at *t* = 1000 ticks and *t* = 1200 ticks. At the earlier point considered in the recovery period of SMEs (*t* = 1000 ticks), mean production capacity is not significantly different between experiments 2 and 3 (*p*-value 0.80067), 2 and 7 (*p*-value 0.20051) and 3 and 7 (*p*-value 0.41519). Similarly, at this point in the recovery period, this is the case when comparing experiments 1 and 5 (*p*-value 0.20051). At time *t* = 1200 ticks in the recovery period, for experiments 2, 3 and 7 the mean production capacity remains not significantly different. In addition, at this point in the recovery period, for experiments 2 and 3 no significant difference is observed with experiments 4 and 6, which themselves are not significantly different.

## 6. Conclusion

A computational modelling and simulation approach has been developed and used to facilitate an evaluation of manufacturing SMEs' operational resilience to extreme flood events. The approach, which couples flood modelling and simulation with agent-based modelling and simulation, provides a means of assessing the relative impact of different types and combinations of flood mitigation measures which SMEs' may implement to limit the damage to their premises, and contents thereof, and disruption to business operations that an extreme inundation event may cause. As a case study, the 2007 extreme flood event in Tewkesbury has been modelled and simulated, and a number of agent-based simulation experiments performed with each corresponding to manufacturing SMEs having implemented different combinations of structural and/or procedural flood mitigation measures. Results suggest that structural flood mitigation measures are more effective than procedural measures in terms of enhancing the operational resilience of manufacturing SMEs. Further, the experiments performed enable an assessment of the relative effectiveness of the different combinations of measures. However, while not included in the approach developed in this research, the financial cost associated with different flood mitigation measures is acknowledged as an important factor for SMEs to consider when deciding which measures could be put in place.

While recognizing the need for careful interpretation of any ABMS results, this research provides the basis of a contribution in terms of informing SMEs of the relative effectiveness of mitigation measures, and combinations thereof, and supporting small business decision-making regarding the implementation of these measures to make their premises more resilient and resistant to flooding. Furthermore, the findings of this research could provide an initial basis for SMEs to consider engaging with (a) UK Government grant schemes to fund particular mitigation measures that improve their property's resilience or resistance to flooding, and (b) the British Insurance Brokers' Association's commercial insurance scheme aimed at improving the ability of this important type of business to find suitable and affordable flood insurance. That is, in terms of resilience and resistance to flooding, this research may encourage better uptake of mitigation measures by SMEs with premises at risk of flooding, which would be recognized in terms of the cost and terms of insurance.

Scope for future work exists in terms of introducing more flood mitigation measures and defining more simulation experiments, with varying combinations of flood mitigation measures. Also, SMEs from different industrial sectors could be considered as well as different geographical locations. In addition, different flood events in terms of severity and duration could be considered, as it is anticipated that these factors will influence the relative difference between operational resilience coefficients for varying combinations of flood mitigation measures. While the case study presented in this paper has focused on the evaluation of SMEs' resilience to a single flood event, it is possible that multiple flood events could occur in relatively quick succession, meaning SMEs would be faced with greater and more complex challenges. Thus, the occurrence of multiple flood events is a potential area of further work. In terms of further development of the model, potential areas for investigation include modelling and capturing the number of insurance claims, loss of staff time and the number of companies going out of business. Given SMEs have such small numbers of employees, a further aspect for inclusion in the model is the rate of return to work post-flood, which will have an impact on these firms' recovery.

## Supplementary Material

Agent-based Model description

## Data accessibility

This article has no additional data available due to it containing confidential information related to SMEs that participated in this research. Also, in agreement with Ordnance Survey regarding MasterMap's Address Layer, the associated data used in this research is not available.
