# Supplementary material for: Evaluating the operational resilience of small and medium-sized enterprises to flooding using a computational modelling and simulation approach: a case study of the 2007 flood in Tewkesbury
Source: Philos Trans A Math Phys Eng Sci. 2020 Feb 17;378(2168):20190210. doi: 10.1098/rsta.2019.0210 (PMC7061973; doi:10.1098/rsta.2019.0210)
Supplement: Agent-based Model description [file rsta20190210supp1.pdf]

AGENT-BASED MODEL DESCRIPTION

This document presents an description of the ABM used in this research providing as much detail as possible given the model contains confidential SME data and information, which we are unable to place in the public domain. The ODD protocol has been as used as a basis for the description of the ABM.

PURPOSE

The purpose of this model is to investigate manufacturing SMEs' immediate response to and short-term recovery from a flood event to evaluate the effectiveness of combinations of flood mitigation measures.

ENTITIES, STATE VARIABLES AND SCALES

Agents

Manufacturing SMEs

Variable name	Brief description
Identifier	Unique identifier for a SME.
SME name	SME's name obtained from OS MasterMap's Address layer.
Business classification code	Code associated with a SME's business classification obtained from OS MasterMap's Address layer.
Business classification name	Name associated with a SME's business classification obtained from OS MasterMap's Address layer.
Easting<br>Northing	Geographic coordinates associated with a SME obtained from OS MasterMap's Address layer.
SME size	Micro, small or medium according to the number of employees.
Clock time list	A list of clock times (half-hour periods) throughout the simulation. This is used to keep a record of the time at which a SME's actions were performed during a simulation.
Simulation ticks list	A list of ticks throughout the simulation; each tick represents a half-hour period.
Days list	A list of days through the simulation; each clock time and simulation tick is associated with a day.
Inundation data list	A list of the water depth(s) at each simulation tick associated with the location of a SME.
Customers' unique identifier list	A list of customers' unique identifiers to enable communication with them when needed (pre and post flood).
Suppliers' unique identifier list	A list of suppliers' unique identifiers to enable communication with them when needed during a simulation (pre and post flood).
Electricians' unique identifier list	A list of electricians' unique identifiers to identifiers to enable communication with them when needed during a simulation (post flood).
Insurance company's identifier	Insurance company's unique identifier to enable communication with it when needed during a simulation (post flood).
Cleaning service providers' unique	A list of cleaning service providers' unique identifiers

Variable name	Brief description
identifier list	to enable communication with them when needed during a simulation (post flood).
Inbox list	A list of messages received from other agents.
Maximum depth of flood water reaches in a SME’s premises	The maximum depth the flood water reaches at a SME’s premises obtained from the inundation data list.
Funds?	True if a SME has funds available.
Compensation?	True if a SME has received financial compensation from its insurer.
Cleaning service provider at premises?	True if the cleaning service provider is present at a SME’s premises.
Allocated employees to clean-up?	True if a SME decides to allocate employees to clean-up the premises rather than requesting a cleaning service provider or accepting a provided cleaning service from its insurer.
Arrival time of cleaning equipment	The arrival time of the necessary cleaning tools acquired for use by a SME’s employees.
Power availability?	True if a SME has power available at its premises.
Electrician at premises?	True if an electrician is present at a SME’s premises.
Trust EA alert?	True if a SME trusts, and so acts on, an EA alert.
Height location of paper documents held above ground level	The height at which paper documents are stored in a SME’s premises.
Total number of employees list	A list of the total number of employees available at an SME’s premises at each simulation tick.
Percentage of machines available list	A list of the percentage of machines available for production at a SME’s premises at each simulation tick.
Proportion of raw materials on the ground list	A list of the proportion of raw materials kept at ground level at a SME’s premises at each simulation tick.
Proportion of raw materials located above ground level list	A list of the proportion of raw materials located above ground level at a SME’s premises at each simulation tick.
Height location of raw materials held above ground level	The height above ground at which raw materials are stored in a SME’s premises.
Raw materials available list	A list of the total quantity of raw materials available at a SME’s premises for production at each simulation tick.
Maximum capacity of raw material that can be stored above ground level	Maximum capacity of raw materials that can be stored above ground level at a SME’s premises.
Production capacity list	A list of the percentage of production capacity at a SME’s premises at each simulation tick.
Proportion of products on the ground list	A list of the proportion of products kept at ground level at a SME’s premises at each simulation tick.
Proportion of products located above ground level list	A list of the proportion of products located above ground level at a SME’s premises at each simulation tick.
Height location of products held above ground level	The height above ground at which products are stored in a SME’s premises.
Products manufactured list	A list of the total quantity of products manufactured at a SME’s premises at each simulation tick.
Maximum capacity of products that can be stored above ground level	Maximum capacity of products that can be stored above ground level at a SME’s premises.
Raise the level at which paper documents are kept	True if paper documents are kept at a raised location at a SME’s premises.
Raise the level at which raw materials	True if a proportion of raw materials are stored at a

Variable name	Brief description
are stored	raised location at a SME’s premises.
Raise the level at which products are stored	True if a proportion of products are stored at a raised location at a SME’s premises.
Keep sandbags on site to seal doorways	True if sandbags are available at an SME’s premises.
Paint exterior of building with waterproofing substances, deploy floodgates and airbrick covers	True if the exterior of a SME’s premises has been painted with waterproofing substances, and floodgates and airbrick covers have been deployed.
Maintain an electricity generator?	True if a SME maintains an electricity generator at its premises?
Raise the level at which machines are located?	True if machines are raised above ground level (on platforms) at a SME’s premises.
Raise the level at which electrical sockets and consumer boards are located?	True if electrical sockets and consumer boards are at raised locations at a SME’s premises.
Install flood-resilient flooring?	True if flood-resilient flooring is installed at an SME’s premises.
Install anti-backflow valves?	True if anti-backflow valves are installed at an SME’s premises.
Maintain/use sump pumps?	True if a SME has a sump-pump system available to remove flood water from its premises.
Store all documentation electronically and maintain backups of them?	True if a SME holds all documentation electronically and maintain backups of them.
Prepare a package of contacts (e.g. customers, suppliers, insurance company, contractors)?	True if a SME holds a package of all contacts.
Prepare and maintain an emergency plan for business continuity?	True if a SME maintains an emergency flood plan for business continuity.
Perform emergency flood exercises?	True if a SME’s employees regularly perform an emergency flood exercise.
Display flood plan instructions?	True if a SME displays its emergency flood plan.
Maintain an emergency financial reserve?	True if a SME maintains an emergency financial reserve.
Register for Environment Agency (EA) flood alerts and warnings?	True if a SME has registered for EA alerts and warnings.
Hold comprehensive insurance cover?	True if a SME holds comprehensive insurance.
Have pre-existing mutual aid partner(s)?	True if a SME has a pre-existing agreement(s) with mutual aid partner(s).
Identify mutual aid partner(s)?	True if a SME identifies a mutual aid partner(s) during a simulation.
Request mutual aid?	True if a SME request mutual aid.

Table 1: Variables of manufacturing SMEs

Cleaning service providers

Variable name	Brief description
Identifier	Unique identifier for the cleaning service provider modelled.
Service type	Cleaning service provider
Status	Available or busy.
Inbox list	A list of messages received from other agents (SMEs or insurance companies).
Senders' unique identifier list	A list of the senders' unique identifiers to enable communication with them when needed during a simulation (post flood).
Teams' status list	A list of the status for every team within the cleaning-service provider signifying the current status, i.e. available or busy.
Number of teams	The number of teams making up the cleaning service provider.
Number of staff in each team	The number of staff in each team within this cleaning service provider.
Insurance companies' unique identifier list	A list of insurance companies' unique identifiers that have a contract with this cleaning service provider.
SMEs' unique identifier list	A list of SMEs' unique identifiers that have a contract with this cleaning service provider.

Table 2: Variables of cleaning service providers

Customers and suppliers

Variable name	Brief description
Identifier	Unique identifier for the agent modelled.
Service type	Customer or supplier.
Inbox	A list of messages received from SME agents.
Senders' unique identifier list	A list of the SME senders' unique identifiers to enable communication with them when needed during a simulation (pre and post flood).
SMEs' unique identifier list	A list of SMEs' unique identifiers that have a contract with this customer or supplier.
Schedule list	A list of customer delivery times or supplier dispatch times.

Table 3: Variables of customers and suppliers

Electrical contractors

Variable name	Brief description
Identifier	Unique identifier for the agent modelled.
Service type	Electrical contractor.
Status	Available or busy.
Inbox	A list of messages received from SME agents.
Senders' unique identifier list	A list of the SME senders' unique identifiers to enable communication with them when needed during a simulation (post flood).

Table 4: Variable of electrical contractors

Environment Agency (EA)

Variable name	Brief description
Identifier	Unique identifier for the agent modelled.
Name	The Environment Agency.
Clock time list	A list of clock times (half-hour periods) throughout the simulation.
Simulation ticks list	A list of ticks throughout the simulation; each tick represents a half-hour period.
Days list	A list of days through the simulation; each clock time and simulation tick is associated with a day.
Inundation data list	A list of the water depth(s) at each simulation tick associated with the location of a SME.
Registered SMEs' unique identifiers list	A list of SMEs' unique identifiers that have registered for EA alerts and warnings.

Table 5: Variable of Environment Agency

Insurance companies

Variable name	Brief description
Identifier	Unique identifier for the agent modelled.
Service type	Insurance company
Inbox	A list of messages received from SME agents.
Senders' unique identifier list	A list of the senders' unique identifiers to enable communication with them when needed during a simulation (post flood).
SMEs' unique identifiers list	A list of SMEs' unique identifiers that are insured with this insurance company.
Cleaning service providers unique identifiers list	A list of cleaning service providers' unique identifiers that have a contract with this insurance company.

Table 6: Variables of insurance companies

Mutual aid partners

Variable name	Brief description
Identifier	Unique identifier for a mutual aid partner.
Mutual aid partner name	Mutual aid partner's name obtained from OS MasterMap's Address layer.
Business classification code	Code associated with a mutual aid partner's business classification obtained from OS MasterMap's Address layer.
Business classification name	Name associated with a mutual aid partner's business classification obtained from OS MasterMap's Address layer.
Easting	Geographic coordinates associated with a mutual aid partner obtained from OS MasterMap's Address layer.
Northing	
Mutual aid partner size	Micro, small or medium according to the number of employees.
Clock time list	A list of clock times (half-hour periods) throughout the simulation.
Simulation ticks list	A list of ticks throughout the simulation; each tick represents a half-hour period.
Days list	A list of days through the simulation; each clock time and simulation tick is associated with a day.
Inbox list	A list of messages received from other agents.
Percentage of machines allocated to SME seeking mutual aid list	A list of the percentage of machines allocated to a SME seeking mutual aid to carry out production at the mutual aid partner's premises at each simulation tick.
Raw materials available list	A list of the total quantity of raw materials available at a mutual aid partner's premises for production at each simulation tick.
Production capacity list	A list of the percentage of production capacity at a mutual aid partner's premises at each simulation tick.
Products manufactured list	A list of the total quantity of products manufactured at a mutual aid partner's premises at each simulation tick.

Table 7: Variables of mutual aid partners

One time step, or simulation tick, represents a half-hour period, which corresponds with the time between flood depths given in the inundation data for each location in the modelled geographic environment. Simulations were run representing approximately 45 working days (i.e. 2132 simulation ticks). The flood event was simulated as described in the accompanying paper. In the area of Tewkesbury considered, 692 SMEs were identified; 92 of these businesses were manufacturing SMEs with 16 of them being flooded as a result of the inundation event modelled.

PROCESS OVERVIEW AND SCHEDULING

The 'process overview and scheduling' element of the ODD protocol aims to present the time when the state variables are updated. However, due to the large size of the model, the process overview is presented as a list. This is in accordance with Grimm et al. (2017) in which it is indicated that it is preferable to demonstrate large models that consist of a repeated sequence of actions as a list of actions. Thus, at each time step (i.e. half-hour period) the following processes occur, in which bold font is used to represent the implemented actions within each of the agents modelled. Please note that Figure 1 indicates the interaction between SME agents and other agents.

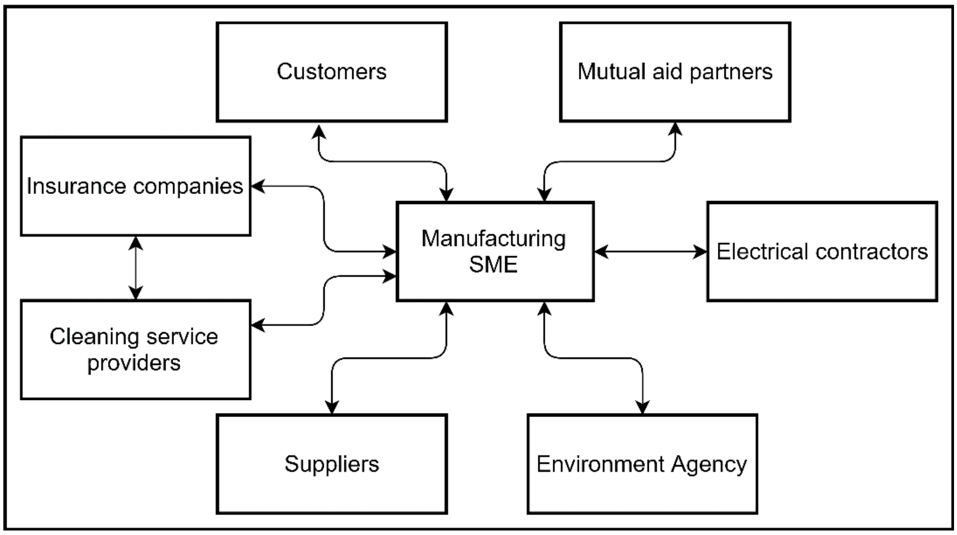

Figure 1: Interactions between SMEs and their related organizations

Environment Agency

The EA agent send alerts and warnings to the registered SMEs at intervals prior to the onset of the flood event.

Manufacturing SMEs

For manufacturing SMEs, the **record information** action will be executed initially to update the simulation's current information, such as list of days, list of clock times, and list of simulation ticks. Furthermore, the **check inbox** action will be executed to check if a flood alert or warning has been received from the EA, or if any new messages have been received from other agents, i.e. suppliers and customers. If no alert or warning is received, SMEs will execute the **production** action. However, if an alert or warning is received, the **emergency** action will be executed.

In relation to the **production** action, this includes a range of actions such as the (i) employee **allocation strategy** action, which decides how many employees will be allocated to work on production or other behaviours if needed, and if applicable (ii) the **duration** action, which updates the duration of related behaviours. For the **emergency** action, this triggers the **allocation strategy** action and the **behaviour** action, which enacts the potential pre-flood behaviours as presented in
Figure 2.

Implementing the **production** and the **emergency** actions will update the following state variables: production capacity; products manufactured; raw materials available.

In relation to the **behaviour** action, the duration associated with each behaviour enacted will be updated as it is undertaken at each simulation tick.

Post-Flood Behaviours

The pre-flood behaviours, which are indicated in Figure 2, are enacted either concurrently or sequentially according to the number of employees available.

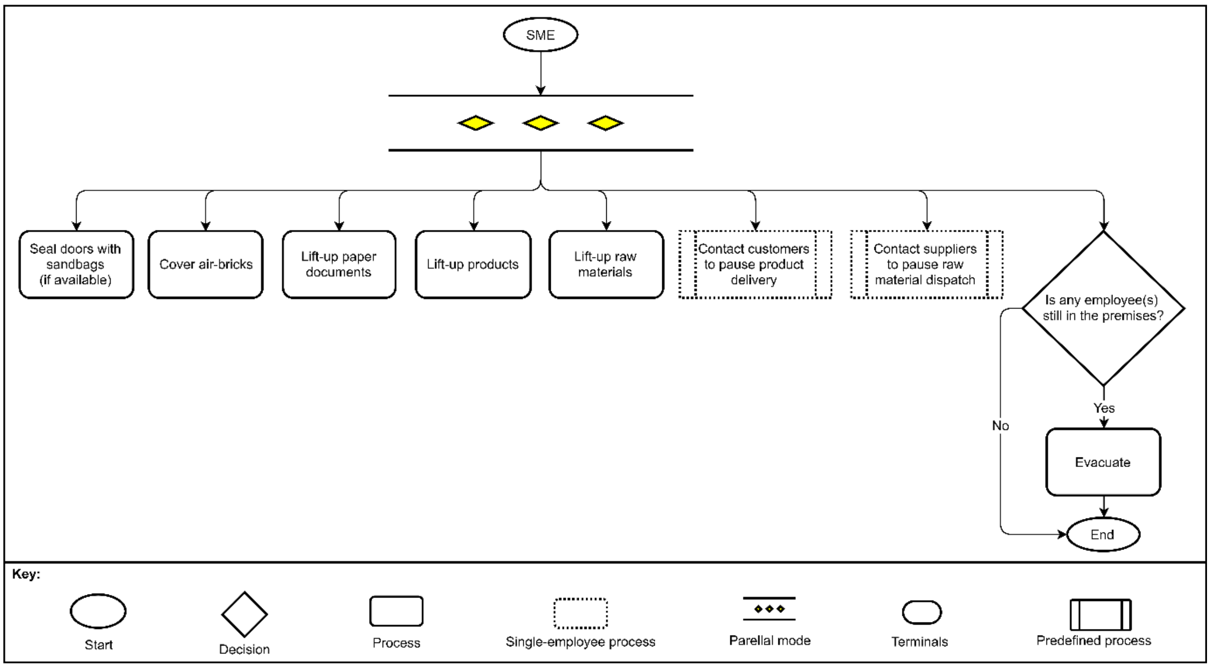

Figure 2: Pre-flood behaviours

To follow, pseudo code of the behaviours indicated in Figure 2 is presented.

- **Seal doors with sandbags**

```
if 'maintained sandbags on site' = true
{
  if 'installed floodgates at doorways' = false
  {
    do Seal doors with sandbag
    update the following attributes:
    'duration of placing sandbags at doorways'
    'completion of placing sandbags at doorways'
  }
}
```

- **Cover air-bricks**

```
if 'EA alerts' = true  or 'EA warnings' = true
{
  do Cover air-bricks
  update the following attributes:
  'duration of covering air-bricks'
  'completion of covering air-bricks'
}
```

- **Lift-up paper documents**

```
if 'raised the locations at which documents are stored' = false
{
  do Lift-up paper documents
```

```
   update the following attributes:
   ‘duration of lifting-up paper documents’
   ‘location height of paper documents’
   ‘completion of lifting-up paper documents’
}
```

- **Lift-up products**

```
if ‘quantity of products available’ > 0
{
   if ‘storage of products’ < ‘maximum capacity of storage of products’
   {
     do Lift-up products
     update the following attributes:
     ‘duration of lifting-up products’
     ‘location height of products’
     ‘completion of lifting-up products’
   }
}
```

- **Lift-up raw materials**

```
if ‘quantity of raw materials on the ground’ > 0
{
  if ‘raw materials above ground space’ < ‘maximum capacity can be placed above ground’
  {
    do Lift-up raw materials
    update the following attributes:
    ‘duration of lifting-up raw materials’
    ‘completion of lifting-up raw materials’
  }
}
```

- **Contact customers to pause product delivery**

```
do Contact customers to pause product delivery
update the following attributes:
'duration of contacting customers to pause product delivery'
'completion of contacting customers to pause product delivery'
```

- **Contact suppliers to pause raw material dispatch**

```
do Contact suppliers to pause raw material delivery
update the following attributes:
'duration of contacting suppliers to pause raw material delivery'
'completion of contacting suppliers to pause raw material delivery'
```

Post-Flood Behaviours

After evacuation, SMEs will be affected by the flood event according to the ‘inundation data’ associated with every individual SME; the degree to which an SME is affected will depend on the flood mitigation measures it has in place.

When the water recedes from an SME premises, the **recovery** action will be invoked to execute the **behaviour** action, which will enact the potential post-flood behaviours (see Figure 3 and 4). Following this, the **allocation strategy** action will be invoked to allocate the appropriate number of employees to work on production and other behaviours. As indicated in the pre-flood behaviours, the duration associated with each behaviour enacted will be updated as it is undertaken at each simulation tick.

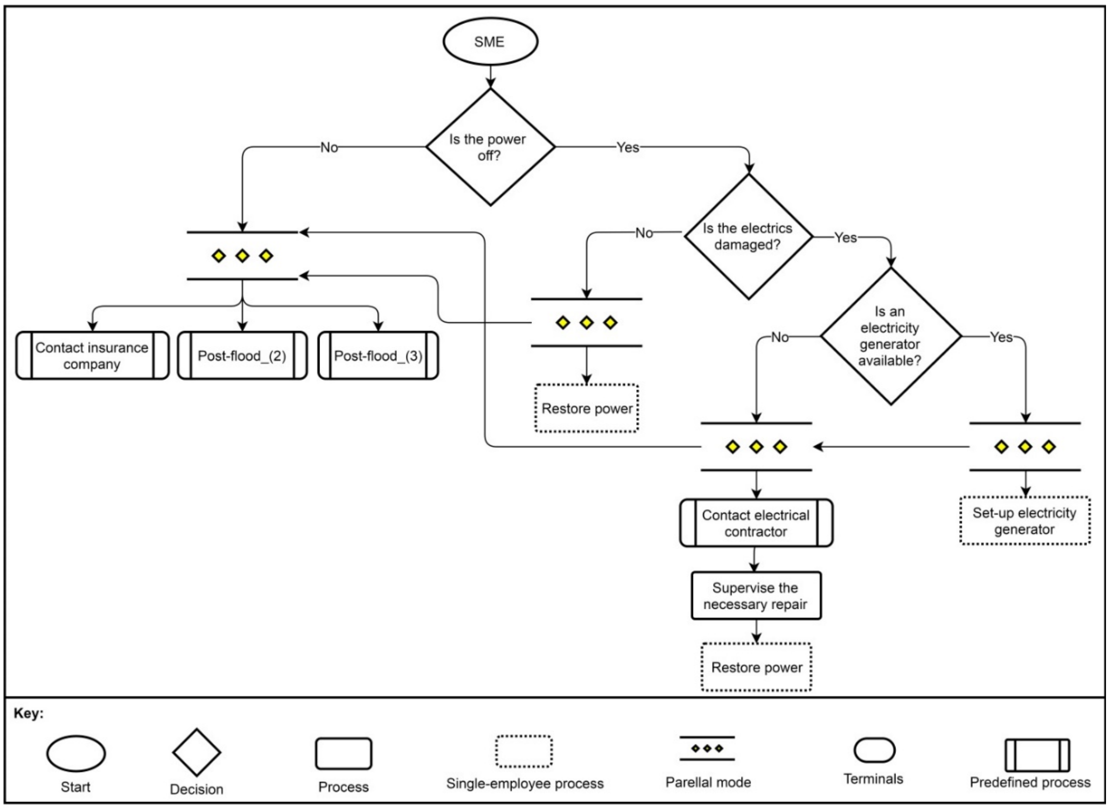

Figure 3: Post-flood behaviours (1)

Pseudo-code of the behaviours indicated in Figure 3 is presented below.

- **Contact insurance company**

```
if 'insurance' = true
{
  do Contact insurance company
  update the following attributes:
  'duration of contacting insurance company'
  'duration of supervising cleaning service provider'
  'cleaning service provider at premises'
  'compensation time'
}
```

- **Contact electrical contractor**

```
if 'funds' = true or 'compensation' = true
{
  if 'maximum level of water depth in a premises' > 0
  {
    if 'maximum level of water depth in a premises' >= 'level of sockets'
    {
      do  Contact electrical contractors
      update the following attributes:
      'duration of contacting electrical contractor'
      'duration of supervising power repair'
      'completion of contacting electrical contractor'
      'electrician at premises'
    }
  }
```

```
}
```

- **Supervise the necessary repair**

```
if 'electrician at premises' = true
{
  do Supervise the necessary repair
  update the following attributes:
  'duration of supervising power repair'
  'duration of supervising power repair'
  'completion of contacting electrical contractor'
  'electrician at premises'
}
```

- **Restore power**

```
if 'maximum level of water depth in a premises' = 0 or 'completion repair power' = true
{
  do Restore Power
  update the following attributes:
  'duration of restoring power'
  'power availability'
  'completion of restoring power'
}
```

- **Set-up electricity generator**

```
if 'maintain an electricity generator on site' = true
{
  if 'power availability' = false
  {
    do Set-up electricity generator
    update the following attributes:
    'duration of setting-up electricity generator'
    'completion of setting-up electricity generator'
    'power availability'
  }
}
```

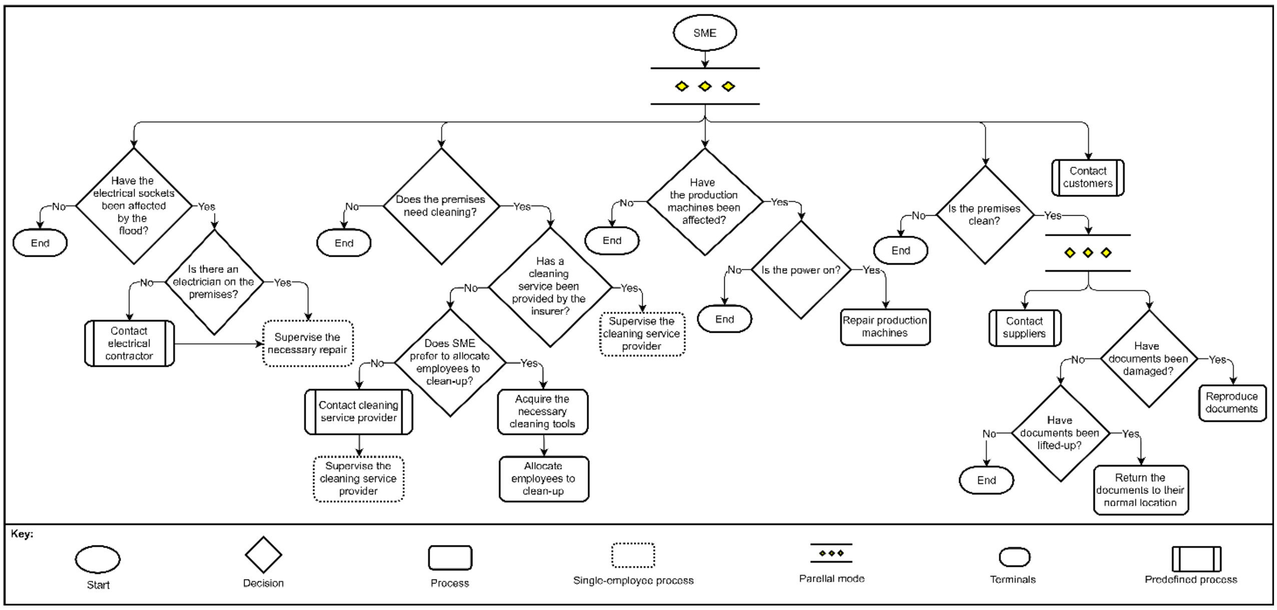

Figure 4: Post-flood behaviours (2)

Pseudo-code of the behaviours indicated in Figure 4 is presented below.

- **Contact cleaning service provider**

```
if ’funds’ = true or ‘compensation’ = true
{
  if ‘insurance type’ = basic
  {
    if ‘allocated employees to clean up’ = false
    {
      if ‘maximum level of water depth in a premises’ > 0
      {
        do Contact cleaning service provider
        update the following attributes:
        ‘duration of contacting cleaning service providers’
        ‘cleaning service providers at premises’
        ‘completion of contacting cleaning service providers’
      }
    }
  }
}
```

- **Supervise the cleaning service provider**

```
if ‘cleaning-service providers at premises’ = true
{
  do supervise the cleaning contractors
  update the following attributes:
  ‘duration of supervising cleaning service’
  ‘completion of clean-up’
  ‘cleaning-service providers at premises’
}
```

- **Acquire the necessary cleaning tools**

```
if ’funds’ = true or ‘compensation’ = true
{
  if ‘allocated employees to clean up’ = true
  {
    if ‘maximum level of water depth in a premises’ > 0
    {
      do Acquire the necessary cleaning tools
      update the following attributes:
      ‘duration of acquiring the necessary cleaning equipment’
      ‘arrival time of cleaning equipment’
      ‘completion of acquiring the necessary cleaning equipment’
    }
  }
}
```

- **Allocate employees to clean-up**

```
if ‘allocated employees to clean up’ = true
{
  if ‘arrival time of cleaning equipment’ >= the current time step ‘tick’
  {
    do Allocate employees to clean-up
    update the following attributes:
    ‘duration of clean-up'
```

```
    'completion of clean-up'
  }
}
```

- **Repair production machines**

```
if 'power availability' = true
{
  if 'completion of setting-up electricity generator' = true
  {
    if 'maximum level of water depth in a premises' > 'the location of at which production machines are placed'
    {
      do Repair production machines
      update the following attributes:
      'duration of repairing production machines'
      'percentage of machines available'
    }
  }
}
```

- **Contact customers**

```
if 'power availability' = true
{
  if 'completion of clean-up' = true
  {
    if 'quantity of products available' > 0
    {
      do Contact customers
      update the following attributes:
      'duration of contacting customers to resume product delivery'
      'completion of contacting customers to resume products delivery'
    }
  }
}
```

- **Contact suppliers**

```
if (maximum level of water depth in a premises' > 0 and 'completion of clean-up' = true) or 'maximum level of water depth in a premises' = 0
{
  do Contact suppliers
  update the following attributes:
  'duration of contacting suppliers to resume raw materials dispatch'
  'completion of contacting suppliers to resume raw materials dispatch'
}
```

- **Return paper documents to normal location**

```
if 'completion of lifting-up paper documents' = true
{
  if 'completion of clean-up'  = true
  {
    do Return paper documents to normal location
    update the following attributes:
    'duration of returning documents to normal location'
    'location height of documents'
    'completion of returning documents to normal location'
  }
```

```
}
```

- **Reproduce documents**

```
if 'maximum level of water depth in a premises' > 'location height of paper documents'
{
  do Reproduce documents
  update the following attributes:
  'duration of reproducing documents'
  'location height of paper documents'
  'completion of reproducing documents'
}
```

After implementing pre- and post-flood behaviours, the **production** action will be invoked. This will update a set of attributes including production capacity. All the aforementioned processes occur at each simulation tick within the model.

Suppliers and customers

As indicated in Figure 5, SMEs interact with suppliers and/or customers. In preparing for a particular flood event, SMEs' behaviours include contacting their suppliers and customers in order to suspend manufacturing raw material and product dispatch. Subsequently, once a SME is in a position to recommence production for manufacturing (a) its supplier is contacted to resume raw material delivery, and (b) customers are informed of the resumption of product dispatch.

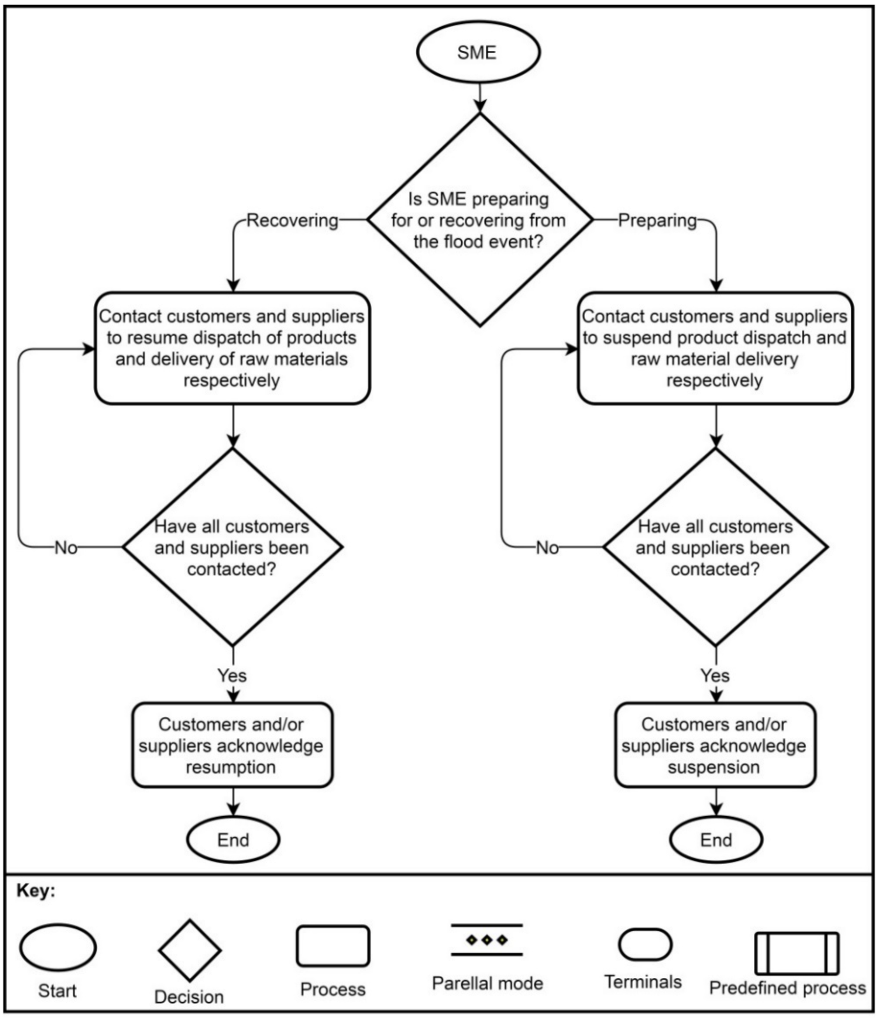

Figure 5: Suppliers and customers

Suppliers interact with SMEs at regular intervals to provide them with raw materials. Within this agent, at each simulation tick, the **check inbox** action is implemented; if the inbox is empty, the **dispatch** action will be executed to continue the dispatch of raw materials if due. However, if a new message was received, the message will be passed to the **interpret message** action, which interprets the received messages along with the sender details such as ‘sender unique identifier’. Subsequently, the appropriate action will be triggered. If the interpreted message indicates a despatch suspension due to the flood event, then the **suspension** action will be executed to update the dispatch schedule list. In addition, the **send message** action will be invoked to respond to the sender (SME) appropriately. If the received message indicates despatch resumption, the **resumption** action will be invoked to update the dispatch schedule list.

The interaction between SMEs and customers is similar to that described for suppliers; however, rather than dispatch of raw materials the focus is on the delivery of products.

Cleaning Service Providers

Cleaning service providers are businesses that interact with manufacturing SMEs and insurance companies. For the latter, cleaning service providers could have a pre-defined contract with insurance companies to provide such a service to insured SMEs if required. SMEs with comprehensive insurance receive assistance in the form of a cleaning service in addition to financial compensation.
At each simulation tick, cleaning service providers execute the **check inbox** action to check if any new messages have been received. If so, they will initially forward the received message(s) to the **interpret message** action, which will interpret the received message along with the sender information (i.e. ‘sender unique identifier’). Also, the check **team availability** action will be executed to inspect if any cleaning team of this service provider is available. If so, the **generate time** action will be invoked to determine a time for sending this team and thus the ‘time to send team’ attribute will be updated. Subsequently, the cleaning service provider will execute the **send message** action to reply to the sender.

Insurance Company

Insured SMEs with comprehensive cover will receive, via its insurance company, assistance from a cleaning service provider in addition to receiving financial compensation. In contrast, SMEs with basic insurance cover will only receive the latter.

As indicated in Figure 6, if the insurance company is contacted by an SME, a loss adjuster will be sent within three days to assess the damage and produce a report followed by payment being released within five working days of the visit. During this period, SMEs may choose to allocate their own employees to work on post-flood behaviours, such as clean-up the premises rather than waiting for the cleaning service provided by the insurance to arrive.

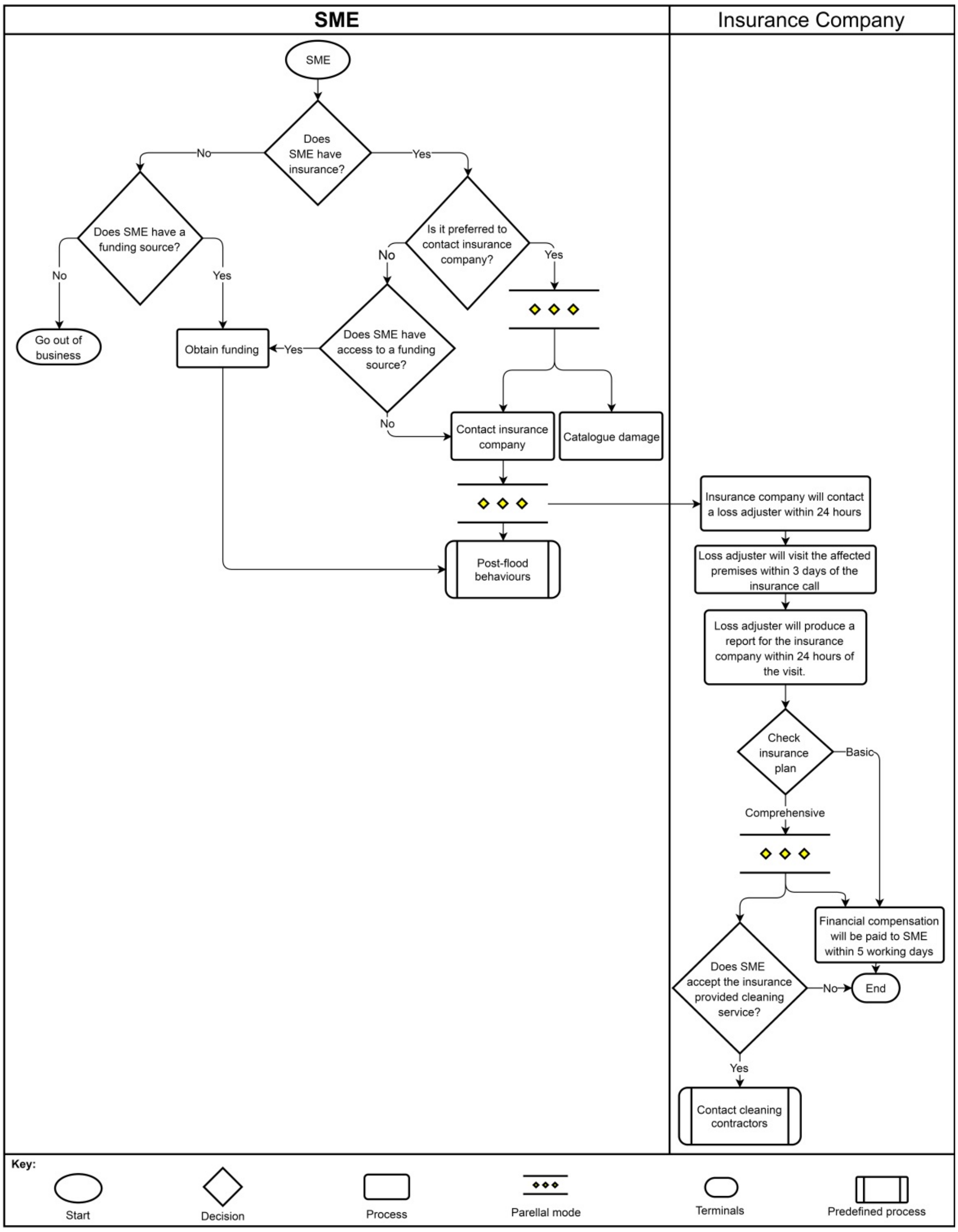

Figure 6: Insurance companies

At each simulation tick, the insurance company executes the **check inbox** action along with any other scheduled events, such as **pay compensation**. If a new message is received, the message will be passed to the **interpret message** action that interprets the received text along with the sender details (i.e. ‘sender unique identifier’ and ‘SME unique identifier’). In the case of comprehensive insurance, the insurer will invoke a **contact cleaning service provider** action that will send a message to the cleaning- service provider it has a contract with along with the SME’s details. Subsequently, the insurer will execute the **compensation** action for both the comprehensive and basic insured SMEs. This action will generate a time to deposit compensation to the affected SME and thus ‘compensation time’ will be updated and passed to the **pay compensation** action.

Electrical Contractors

These contractors interact with SMEs. In terms of the interaction, the contractors interact with SMEs via messages to attend at an SME's premises after a flood event. This is to assist with SMEs' to return to the normal operation (pre-flood). As shown in Figure 7, SMEs must have funds available to request such services. Funds can be derived from three defined sources: (i) insurance compensation, or (ii) emergency financial reserves. Insurance compensation represents the most common source of funds, but an SME may have to wait for longer period to receive it. In terms of emergency financial reserves, SMEs can proceed with the short-term recovery process immediately. With sufficient funds available, SMEs will contact the electrical contractors.

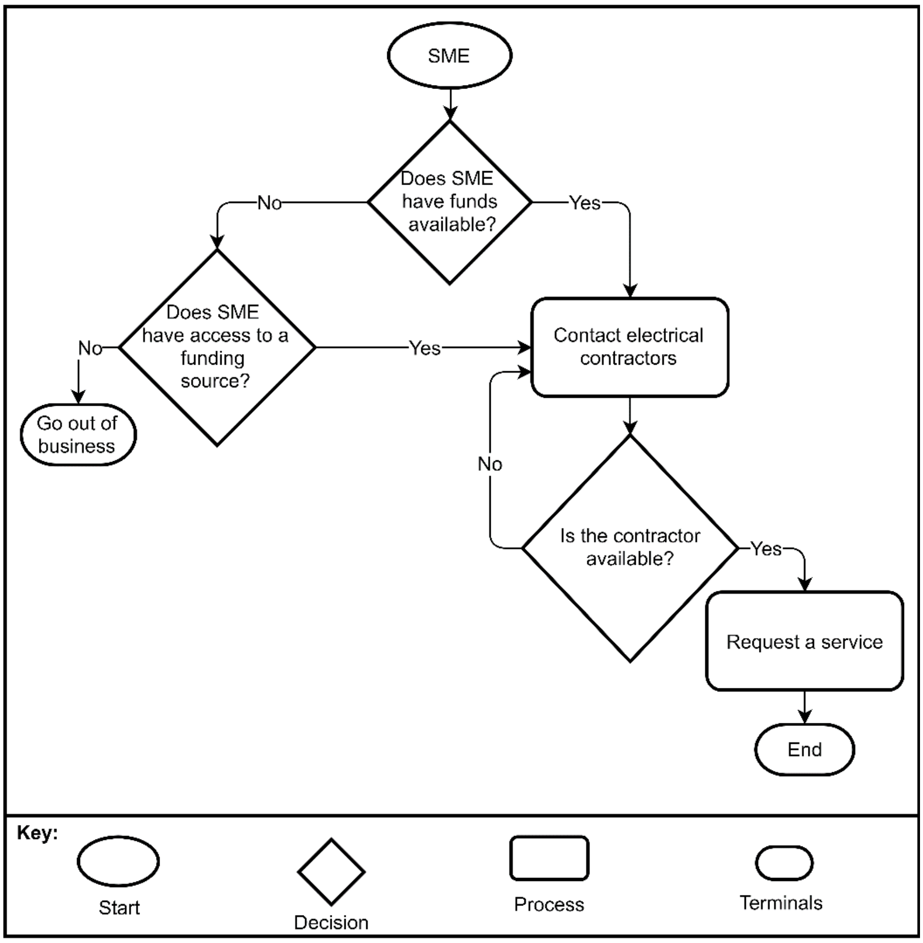

Figure 7: Electrical contractors

At each simulation tick, electrical contractors run the **check inbox** action along with the **go to SMEs** action. If a new message is received, the received message will be passed to the **interpret message** action that interprets the received text along with the sender details (i.e. 'sender unique identifier'). Electrical contractors will check their status and reply. If the status is free, they will update the 'time to go' attribute and then pass it to the **go to SMEs** action, which is checked at each simulation tick. Also, electricians will invoke the **send message** action to respond to the affected SME(s). However, if the status is 'busy', electricians will respond to the affected SME appropriately.

DESIGN CONCEPTS

Basic Principles

The model investigates the effect of flood mitigation measures that can be put in place by manufacturing SMEs to maintain business continuity and speed-up short-term recovery from flooding. This modelling/simulation approach consists of three main components: (i) flood-event modelling and simulation; (ii) a modelled geographical environment (MGE); and (iii) agent-based modelling and simulation. First, flood-event simulation represents the input (inundation) data that feeds into the MGE component, which provides a common physical environment for the flood-event modelling and simulation and the agent-based modelling and simulation. Specifically, the MGE combines Ordnance Survey (OS) MasterMap® data (i.e. Topography, Integrated Transport Network (ITN), and Address layers) with the input from the flood-event modelling/simulation to enable the identification of flooded and non-flooded organisations. Consequently, SMEs from the manufacturing sector are modelled. In addition, SME-related organisations are also modelled to support SMEs' operation and/or recovery. In terms of manufacturing SMEs, they are modelled as agents with sets of attributes, behaviours, and flood mitigation measures. Production capacity is computed at each simulation tick for every manufacturing SME modelled to determine their performance throughout the simulation.

Emergence

Not applicable to this model.

Adaptation

Manufacturing SMEs that have implemented flood mitigation measures tend to respond better and recover more quickly than SMEs implementing none of them. For example, a manufacturing SME that has machines raised on platforms/plinths above the level at which the flood water reaches in its premises will not need to allocate employees to repair them. Similarly, SMEs that have registered for EA alerts and warnings may enact pre-flood behaviours, which will potentially reduce the damage caused by flood water entering its premises.

Objectives

Manufacturing SMEs want to minimise production capacity loss throughout the period simulated by implementing a number of or all of the flood mitigation measures. The Environment Agency wants to help minimise disruption to SMEs by sending alerts and warnings to all those registered. Suppliers aim to provide manufacturing SMEs with raw materials so as to enable production at a SME. Insurance companies aim to assist SMEs with financial compensation for any flood damage and, if covered sufficiently, send a cleaning service provider to attend its premises. Cleaning-service providers aim to clean-up SMEs' premises when requested, thus accelerating recovery. Electrical contractors aim to provide the necessary repairs at a SME's premises when requested in order to accelerate recovery.

Learning

The model focuses on investigating how SMEs' behaviours and flood mitigation measures, and combinations of them, impact post-flood short-term recovery. Thus, within a simulation, SMEs do not learn from the other agents.

Prediction

This model does not predict future conditions SMEs will experience.

Sensing

SME agents within the model save all information related to them, including the flood mitigation measures in place, which influences the choice of behaviours they enacted and the duration of these behaviours. Recording all information related to agents allows an analysis of production capacity throughout the simulation period to be carried out, which then enables the operational resilience coefficient to be determined.

Interaction

Manufacturing SMEs interact with other organisations via messages. As indicated in Table 8, SMEs can interact with organisations pre and/or post flood.

Organisations	Period of interaction	Function
**Suppliers**	Pre- and post-flood	Provide both manufacturing SMEs with raw material.
**Customers**	Pre- and post-flood	Take delivery of products from manufacturing SMEs.
**Environment Agency**	Pre-flood	Send alerts and warnings to SMEs that have registered for these services.
**Insurance companies**	Post-flood	Provide insured SMEs with assistance in the form of a cleaning service, if insured sufficiently, in addition to financial compensation.
**Electrical contractors**	Post-flood	Provide the necessary repairs.
**Cleaning service providers**	Post-flood	Provide cleaning service.
**Mutual aid partners**	Post-flood	Enable continuity in production.

Table 8: Information related to SME-related organisations

Stochasticity

Each of a SME's behaviours (please see Table 9) has an associated duration range obtained from transcripts of semi-structured interviews with SMEs having experience of flooding or being at risk of flooding. In addition, the duration of post-flood behaviours can be affected by the flood mitigation measures implemented by a manufacturing SME.

Number	Pre and post-flood behaviours
1	Seal doorways with sandbags
2	Cover air-bricks
3	Lift-up paper documents
4	Lift-up products
5	Lift-up raw materials
6	Evacuate
7	Contact insurance company
8	Contact electrical contactor
9	Supervise the necessary repair (by electrical contractor)
10	Restore power
11	Set-up electricity generator
12	Contact cleaning contractor
13	Supervise cleaning contractor

Number	Pre and post-flood behaviours
14	Acquire the necessary cleaning tools
15	Allocate employees to clean-up
16	Repair production machines
17	Contact suppliers to resume their delivery of raw materials
18	Contact customers to inform of resumption of SME dispatch of products
19	Return documents to their normal location
20	Reproduce documents

Table 9: SME pre and post-flood behaviours

Collectives

Not applicable.

Observation

In a simulation, data are collected on a half-hourly basis for each manufacturing SME. Based on values related to the availability of employees, machines, raw materials and power, the key manufacturing SME performance metric, namely production capacity (at each simulation tick) is determined.

INITIALIZATION

This model consists of a number of types agents representing different types of organisation; all agents are created at the beginning of the simulation. At the same time, agent variables are assigned. Most variables are set the same for each manufacturing SME agent; however, some are randomly assigned such as number of employees, behaviour durations (with a specified range).

INPUT

In terms of external sources, the model uses input data from Ordnance Survey MasterMap© (Topography layer, Address layer and Integrated Transport Network™ layer). In addition, the model uses inundation data provided from flood modelling and simulation.

SUB-MODELS

Environment Agency (EA)

For this agent, the **Watch-flood** action is scheduled to run at each simulation tick. The pseudo code below presents a simplified overview process of the **watch-flood** action that sends alerts and warnings to the registered SMEs.

```
if (tick = 1)
  flood commencing time = updated flood times();
else
{
  if (tick = flood commencing time – 36 hours) then
  {
   implement send alerts to SMEs
  }
    if  (tick = flood commencing time – 3 hours) then
```

```
  {
   implement send warnings to SMEs
  }
}
```

In relation to the pseudo code, at the first tick, the update flood time method will be invoked to update the flood commencing time. Next, **send alerts to SMEs** action is implemented 36 before the flood. In terms of the flood warnings, they are sent three hours before the flood occurs.

Manufacturing SME

For manufacturing SME agents, the production capacity is used as the performance metric. Production capacity is related to availability of raw materials (RM), machines (M), employees (E) and power (P). The relationships between these dynamic attributes are presented in the following pseudo code.

```
if P = 1 then
{
    if raw materials at time t, RM_t ≥ min {E, M} then
    {
        PL= min {E, M} × 100
        RM_{t+1} = RM_t − min {E, M}
    }
    else if RM_t<min {E, M}
    {
        PL = RM_t×100
        RM_{t+1} = 0
    }
}
else if P = 0
{
    PL = 0
    RM_{t+1} = RM_t
}
```

In relation to the pseudo code, for each simulation tick (i) production capacity and (ii) raw materials available at the next tick, are determined depending on whether power is available at the SME's premises or not. For situations in which power is available, and depending on the amount of raw materials available at the current tick, both (i) and (ii) are determined according to the relationship between ratios of employees working on production to total number of employees, and machines available to total number of machines respectively.

Emergency

The **emergency** action is implemented within a manufacturing SME; this action invokes three different actions: (a) **duration,** (b) **allocation strategy,** (c) **behaviours** as presented in the pseudo code below.

```
if tick = 1 then
{
  implement Duration
}
implement Allocation Strategy
Implement Behaviours
```

In relation to the **duration** action, it is executed in the first tick of the simulation to initialize the durations of the behaviours' listed in Table 9.

Allocation Strategy of Employees

The **allocation strategy** action is invoked to allocate the appropriate number of employees to behaviours. Four employee allocation strategies ('*normal*', '*flood is possible*', '*immediate action*', and '*recovery*') are considered in which each of these is implemented in different situations. The '*normal*' strategy is implemented prior to receiving flood alerts, in which all the employees are allocated to work on production respectively. The '*flood is possible*' allocation strategy begins after receiving a flood alert; this may last until the time at which flood warnings are received or a flood commences. In the '*flood is possible*' strategy, a proportion of employees will prepare for the potential event while the rest will work on production. The '*immediate action*' strategy is implemented after receiving a flood warning, at which time employees are allocated to prepare for the potential event before evacuating, or evacuating immediately. During the flood, employees are not at a SME's premises. In the aftermath, the '*recovery*' strategy is implemented in which the main focus is restarting production.

A simplified overview of the allocation strategy action is presented in the following pseudo code.

```
if period = pre-flood then
{
    if alert = false and warning = false then
    {
            implement normal
    }
    if alert = true and warning = false then
    {
        if trust alert = true then
        {
            implement flood is possible
        }
        else if trust alert = false then
        {
            implement normal
        }
    }
    else if warning = true then
    {
        implement immediate action
    }
}
else if period = post-flood then
{
    if post-flood behaviours have not been completed then
    {
        implement recovery
    }
    else if post flood behaviours have been completed then
    {
        implement normal
     }
}
```

In relation to the pseudo code, for each simulation tick, the appropriate strategy is determined depending on whether the period is pre-flood or post-flood. Within the pre-flood period, three

strategies may be invoked: (i) the ‘normal’ strategy, (ii) the ‘flood is possible’ strategy and (iii) the ‘immediate action’ strategy. In the post-flood period, if some of the post-flood behaviours are incomplete, or yet to be enacted, then the ‘recovery’ strategy will be implemented. However, if all the post-flood behaviours have been enacted, the ‘normal’ strategy will be implemented with all employees working on production.
The *flood is possible* allocation strategy allocates one and two employee(s) to the single and multiple employee pre-flood behaviours respectively. At the same time, all employees unallocated to these behaviours work on production. This strategy aims to provide pre-flood behaviours with the minimum possible number of employees required to enact them (see the following pseudo code).

```
for i = 1 to n, where n is the number of the pre-flood behaviours
{
   while the number of employees available at a manufacturing SME premises > 0
   {
     if number of employees available at a manufacturing SME premises > 1, then
     {
         if behaviour requires multi-employee, then
         {
             this behaviour is allocated two employees
             allocated number of employees = 2
         }
         else if behaviour requires a single employee, then
         {
             this behaviour is allocated one employee
             allocated number of employees = 1
         }
     else if number of employees available at a manufacturing SME premises = 1, then
     {
         this behaviour is allocated one employee
         allocated number of employees = 1
     }
     else
     {
         break
     }
     update allocated number of employees
   }
}
```

In relation to the pseudo code, for each simulation tick, every behaviour is allocated employees based on the number of employees available. For each pre-flood behaviour yet to be enacted, and while all employees are yet to be allocated, if the total number of employees available is greater than one, then the appropriate number of employees (i.e. one or two) will be allocated to the behaviour. However, if only one employee is available, then that employee will be allocated to the behaviour under consideration.

The *immediate action* allocation strategy allocates all the available employees to work on non-production behaviours. Employees will evacuate: (i) when all these behaviours are enacted, (ii) at the end of the working day, or (iii) when the flood event occurs. Note that, if the EA alert or warning was not received, then the *immediate action* strategy will not be implemented, and employees will evacuate when the flood event occurs.

The *recovery* allocation strategy ensures that all an SME’s employees are allocated to production if power is supplied to the premises and there are sufficient raw materials and machines available. However, if all production employees are available to work on production but there are some post-

flood behaviours yet to be completed, then one production employee will be allocated to complete those behaviours; the remainder will work on production. Note that if more production employees are available than there are able to work on production, then the surplus of these employees is allocated to work on other behaviours.
